# Metastatic Patterns of Apical Lymph Node and Prognostic Analysis in Rectal and Sigmoid Colon Cancer—A Multicenter Retrospective Cohort Study of 2809 Cases

**DOI:** 10.3390/cancers17142389

**Published:** 2025-07-18

**Authors:** Mingguang Zhang, Fuqiang Zhao, Aiwen Wu, Xiaohui Du, Lei Zhou, Shiwen Mei, Fangze Wei, Shidong Hu, Xinzhi Liu, Hua Yang, Lai Xu, Yi Xiao, Xishan Wang, Qian Liu, on behalf of the Chinese Apical Lymph Node Study Consortium

**Affiliations:** 1 Department of Colorectal Surgery, National Cancer Center/National Clinical Research Center for Cancer/Cancer Hospital, Chinese Academy of Medical Sciences and Peking Union Medical College, Beijing 100021, China; zmgslimshady@163.com (M.Z.); zhaofqncc@163.com (F.Z.); mswbxw@163.com (S.M.); WFZpumch@163.com (F.W.); 2 Key Laboratory of Carcinogenesis and Translational Research (Ministry of Education), Department of Gastrointestinal Cancer, Peking University Cancer Hospital and Institute, Beijing 100142, China; wuaw@sina.com (A.W.); liu_xinzhi@hotmail.com (X.L.); 3 Department of General Surgery, The First Medical Center, Chinese Peoples’ Liberation Army General Hospital, Beijing 100853, China; duxiaohui301@sina.com (X.D.); hushidong2014@163.com (S.H.); 4 Department of Gastrointestinal Surgery, China-Japan Friendship Hospital, Beijing 100029, China; zhoulei1971@hotmail.com (L.Z.); yanghuadw@126.com (H.Y.); 5 Department of General Surgery, Peking Union Medical College Hospital, Chinese Academy of Medical Sciences and Peking Union Medical College, Beijing 100021, China; drxulai@pumch.cn (L.X.); xiaoy@pumch.cn (Y.X.)

**Keywords:** apical lymph node, lymph node metastatic patterns, rectal cancer, sigmoid colon cancer, prognosis

## Abstract

**Background/Objectives**: The metastatic patterns of apical lymph node (ALN) in rectal and sigmoid colon cancer are currently unclear, and there is no consensus on the indications for dissection of ALN. This study aimed to analyze the impact of ALN metastasis on prognosis, determine the metastatic patterns of ALN and provide evidence for indications of ALN dissection in rectal and sigmoid colon cancer. **Methods**: In this multicenter, retrospective cohort study, patients from five centers with stage I-III rectal or sigmoid colon cancer who underwent laparoscopic radical surgery with ALN dissection without neoadjuvant treatment from January 2015 to December 2019 were enrolled. **Results**: Among 2809 patients, the positive rate of ALN was 1.9%. The 5-year overall survival and cancer-specific survival rate for patients with metastatic ALN were 37.5% and 41.0%, respectively. ALN metastasis was the independent risk factor for poor prognosis. Tumor size ≥5 cm (OR = 2.32, 95% CI: 1.30–4.13, *p* = 0.004), signet ring cell cancer/mucinous adenocarcinoma (vs. poor differentiated adenocarcinoma, OR = 0.19, 95% CI: 0.08–0.45, *p* < 0.001; vs. moderate to well differentiated adenocarcinoma, OR = 0.22, 95% CI: 0.11–0.42, *p* < 0.001), T4 stage (OR = 1.93, 95% CI: 1.05–3.55, *p* = 0.034), N2 stage (OR = 8.86, 95% CI: 4.45–17.65, *p* < 0.001) and radiologic evidence of extramural venous invasion (OR = 1.88, 95% CI: 1.03–3.42, *p* = 0.040) were independent risk factors for ALN metastasis. The nomogram model developed by these factors achieved a good predictive performance. **Conclusions**: This research offered insights into the incidence, risk factors, and prognostic significance of apical lymph node metastasis in cases of rectal and sigmoid colon cancer. Additionally, the study furnished empirical support for the criteria guiding ALN dissection. Furthermore, a pragmatic risk assessment model was developed to predict ALN metastasis.

## Simple Summary

The metastatic pattern of apical lymph node (ALN) in colorectal cancer remains unclear. This study aimed to comprehensively evaluate the incidence, clinicopathological risk factors, and prognostic implications of ALN metastasis in patients undergoing surgical resection for rectal and sigmoid colon cancer. Through detailed analysis of a large cohort, we identified key predictors associated with ALN involvement and constructed a clinically applicable nomogram to estimate the probability of metastasis. This predictive model allows for individualized risk assessment and may assist surgeons in optimizing lymphadenectomy strategies, especially in cases where extended lymph node dissection is being considered. By identifying patients at higher risk for ALN metastasis, the model contributes to more informed clinical decision-making and holds potential for improving the precision and effectiveness of lymph node dissection.

## 1. Introduction

The apical lymph node, also referred to as the station 253 lymph node, holds a pivotal position as the third station in the lymph node of the inferior mesenteric artery (IMA) system. This node is crucial in the lymphatic circulation of the sigmoid colon and rectum. It serves as the last barrier to tumor metastasis from the regional to distant areas [1]. The metastasis status of apical lymph node is closely related to prognosis [2,3,4,5]; hence, the strategy for clearing that region is critical in the radical surgical resection of rectal and sigmoid colon cancer.

There remains a contentious debate regarding selecting the level for ligation of the IMA and the necessity of removing the apical lymph node for all cases. The National Comprehensive Cancer Network (NCCN) guidelines do not explicitly state whether there is a need to clear the apical lymph nodes, only emphasizing the requirement to harvest at least 12 lymph nodes [6]. The Japanese Society for Cancer of the Colon and Rectum (JSCCR) guidelines advises that the lymph node around the IMA should be routinely cleaned, except in cases where tumors are confined to the muscular layer and there is no pre-surgical evidence of lymph node metastasis [7].

High ligation (HL) accompanied by apical-node dissection has the potential to enhance the lymph node yield rate. However, this approach may risk compromising the local blood supply [8,9,10] or potentially cause injury to the autonomic nerves surrounding the IMA [11,12]. If the metastatic status of the apical lymph node can be predicted, it would enable precise and selective lymph node dissection, thereby avoiding more invasive surgical interventions. However, the metastatic pattern of apical lymph node in colorectal cancer remains unclear, with significant variations in findings across different studies [2,11,12,13,14]. Therefore, this study was initiated to investigate the metastatic patterns of apical lymph nodes, to assist in predicting their metastatic status preoperatively, thereby providing evidence for determining the extent of lymph node dissection in rectal and sigmoid colon cancer. Additionally, this study further explored the impact of ALN metastasis on prognosis in rectal and sigmoid colon cancer.

## 2. Materials and Methods

### 2.1. Study Design

This study is a retrospective multicenter cohort study, including patients with sigmoid colon cancer, rectosigmoid cancer, and rectal cancer who underwent surgery from January 2015 to December 2019. All participating centers are large tertiary hospitals in China. To minimize the quality differences in surgery across multiple centers, the following requirements were set: (1) surgeons participating in the study must have over 10 years of experience in colorectal surgery; (2) colon cancer surgeries must adhere to the principle of complete mesocolic excision (CME), and rectal cancer surgeries must follow the total mesorectal excision principle (TME); (3) the IMA can be ligated at a high or low level, but D3 radical surgery for tumor must be completed. This includes meticulous cleaning of lymph nodes within the mesentery and the apical lymph node in the area of the root of the IMA, which is then confirmed by both the surgeon and the pathologist regarding the lymph nodes removed during surgery.

Inclusion Criteria:

(1) Histopathologically confirmed as adenocarcinoma, mucinous adenocarcinoma or signet ring cell carcinoma.
(2) The lesion location was in the sigmoid colon and rectum.
(3) Intention-to-treat was curative and apical lymph node was dissected.
(4) Clinicopathological data was completed.

Exclusion Criteria:

(1) Patients with history of colorectal cancer.
(2) Patients underwent neoadjuvant therapy.
(3) Patients with multiple primary colorectal cancers.
(4) Patients with family history of hereditary diseases.
(5) Patients with extra-regional lymph node metastasis.
(6) Patients with distant metastasis.

### 2.2. Data Collection and Analysis

Clinical Data Collection:

Factors such as age, gender, tumor location, pathological type, degree of tumor differentiation, T stage, N stage, TNM stage, tumor size, extramural venous invasion (EMVI), BRAF status and mismatch repair (MMR) status were collected. The TNM staging referred to the 8th edition of the AJCC staging for colorectal cancer. Tumor location was categorized based on colonoscopy reports, radiological image and intraoperative findings into the sigmoid colon, upper rectum (terminal sigmoid colon to the peritoneal reflection) and lower rectum (below the peritoneal reflection).

Development of Predictive Model:

The influencing factors of the ALN metastasis were analyzed. Utilizing the data from center A, B and C as the training set (1964 cases), a nomogram was constructed based on the risk factors for ALN metastasis in univariate logistic regression. Other centers’ data were used as an external validation set (845 cases) to evaluate the effectiveness of the nomogram.

### 2.3. Surgical Procedures

**Figure A1:**
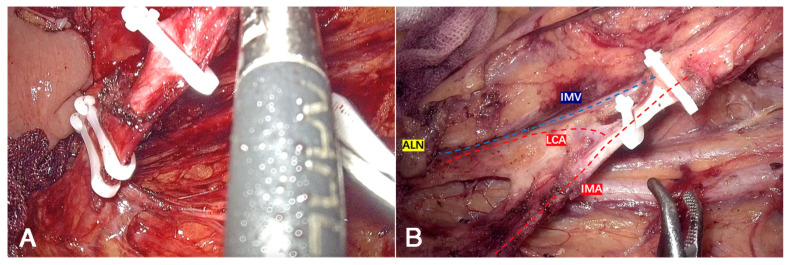
Two different methods of vascular ligation. (**A**) Ligation performed at the origin of IMA with ALN dissection. (**B**) Ligation performed at distally to the origin of the left colic artery with ALN dissection.

For rectal cancer, surgery was performed according to the principle of the TME. For sigmoid colon cancer, the CME principle was applied. During surgery, the root of the IMA was fully exposed, and the ALN was thoroughly dissected, with high ligation of the IMA along the artery (Appendix A Figure A1A); or the bifurcation of the left colic artery was exposed, and the IMA was ligated distally to the origin of the left colic artery, ensuring the dissection of the ALN while preserving the left colic artery (Appendix A Figure A1B). For sphincter-preserving surgery, the tumor was resected with at least 2 cm distally and at least 10 cm proximally, and the specimen was removed through an abdominal auxiliary incision. If the bowel condition was favorable, primary digestive reconstruction could be performed with an end-to-end anastomosis of the sigmoid colon to the rectum, and whether to perform a prophylactic stoma was decided based on the actual situation. If the bowel condition was poor and not suitable for primary anastomosis, Hartmann’s procedure was performed. If the tumor’s location was low and sphincter preservation was not possible, a laparoscopic abdominoperineal resection would be carried out.

### 2.4. Follow-Up and Postoperative Therapy

Follow-up of patients was conducted through telephone and outpatient review. One month after surgical treatment and then every three months, follow-up visits were conducted to assess survival status. This study utilized overall survival (OS) and cancer-specific survival (CSS) to evaluate survival outcomes. OS refers to the duration of time from the surgery to the time of death from any cause or to the last follow-up time. CSS refers to the time from the surgery to death caused solely by the cancer or to the last follow-up time.

Postoperative adjuvant therapy follows the NCCN guidelines. Patients with rectal cancer who are pathologically staged as stage III or stage II with risk factors (such as poor differentiation, mucinous adenocarcinoma or signet ring cell carcinoma, T4, lymphovascular invasion, or perineural invasion) will receive adjuvant radiotherapy and chemotherapy. Patients with sigmoid colon cancer under the same conditions will undergo adjuvant chemotherapy.

### 2.5. Statistical Analysis

SPSS 23.0 and R 4.0.2 statistical software were used to analyze the data. Categorical data were presented as absolute numbers and percentages. Univariate analysis of influencing factors was conducted, with unordered categorical data analyzed using the χ2 test or Fisher’s exact test, and ordered categorical data using the Wilcoxon rank-sum test. Factors statistically significant in univariate analysis were included in a binary logistic regression model for multivariate analysis to identify independent influencing factors. The evaluation of the nomogram was based on the area under the receiver operating characteristic curve and calibration curves. The calibration curves were established based on 1000 bootstrap resamples, calculating the OR and 95% CI. Survival analysis was performed using the Kaplan–Meier method and Log-rank test, while prognostic factors were analyzed using the Cox regression model, with factors significant in univariate analysis included in the multivariate analysis model. A *p*-value of <0.05 was considered statistically significant.

## 3. Results

### 3.1. Clinicopathological Characteristics

A total of 2809 patients were included in this study. Among them, 53 patients were positive for ALN, accounting for 1.9%. The basic clinicopathological information was shown in Table 1. The average age was 61.60 ± 11.19 years. Regarding gender, 1721 patients (61.3%) were male and 1088 patients (38.7%) were female. The tumor locations were distributed among the sigmoid colon (34.4%), the higher rectum (42.7%), and the lower rectum (22.9%). Histological subtype was classified as signet ring cell cancer or mucinous adenocarcinoma in 410 patients (14.6%), poorly differentiated in 494 patients (17.6%) and moderate to well differentiated in 1905 patients (67.8%). The T stage distribution was as follows: T1 in 179 patients (6.4%), T2 in 544 patients (19.4%), T3 in 1233 patients (43.9%), and T4 in 853 patients (30.4%). For the N stage, 1581 patients (56.3%) were at N0, 413 patients (14.7%) at N1, and 815 patients (29.0%) at N2. The TNM stages were stage I for 554 patients (19.7%), stage II for 1027 patients (36.6%), and stage III for 1228 patients (43.7%). Tumor size was less than 5 cm in 2043 patients (72.7%) and more than 5 cm in 766 patients (27.3%). The distribution of BRAF V600 mutation status showed that the majority of patients were wild-type (2624 cases, 93.4%), while mutant BRAF V600 was observed in 185 cases (6.6%). Regarding mismatch repair (MMR) status, proficient MMR (pMMR) was identified in 2577 patients (91.7%), whereas deficient MMR (dMMR) was observed in 232 patients (8.3%). In total, 1950 patients (69.4%) underwent adjuvant chemoradiotherapy or chemotherapy after surgery.

### 3.2. Survival Analysis

**Figure 1:**
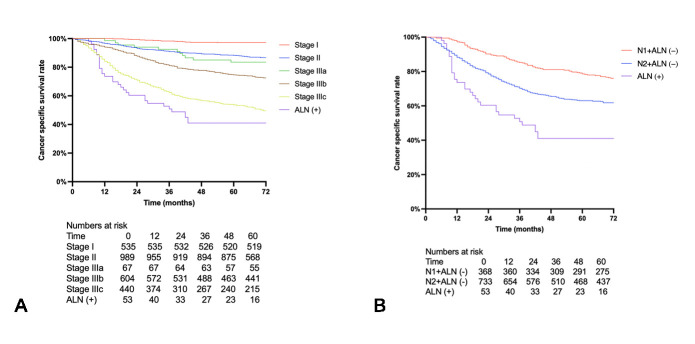
Kaplan–Meier survival curve for patients in different stages. (**A**) Comparing cancer-specific survival among patients with ALN metastasis to those with different TNM stages. ALN-positive patients had the poorest prognosis, significantly worse than patients in stage IIIc (HR 1.57, 95% CI 1.01–2.45, *p* = 0.045). (**B**) Comparing cancer-specific survival among patients with ALN metastasis to those with different N stages. ALN-positive patients had significantly worse prognosis than those in stage N2 (HR 2.42, 95% CI 1.48–3.97, *p* < 0.001).

The total cohort had a median follow-up time of 66 months, with 127 cases lost to follow-up, resulting in a loss to follow-up rate of 4.5%. The 5-year OS and CSS for patients positive for ALN were 37.5% and 41.0%, respectively. Researchers conducted a subgroup analysis based on different TNM stages and found that ALN-positive patients had the poorest prognosis, significantly worse than patients in stage IIIc (HR 1.57, 95% CI 1.01–2.45, *p* = 0.045) (Figure 1A). Further subgroup analysis on the population with lymph node metastasis revealed that ALN-positive patients had significantly worse prognosis than those in stage N2 (HR 2.42, 95% CI 1.48–3.97, *p* < 0.001) (Figure 1B). In the cohort of patients at TNM stage III, Cox regression analysis revealed that ALN metastasis was the independent risk factor of poor prognosis (OS: HR 2.02, 95% CI 1.39–2.95, *p* < 0.001; CSS: HR 2.06, 95% CI 1.40–3.03, *p* < 0.001) (Table 2).

### 3.3. Risk Factors of ALN Metastasis

The univariate analysis results showed that a tumor located in the sigmoid colon or upper rectum (*p* = 0.007), tumor size ≥5 cm (*p* < 0.001), histological subtype of mucinous adenocarcinoma or signet ring cell carcinoma (*p* < 0.001), T4 stage (*p* < 0.001), N2 stage (*p* < 0.001) and positive EMVI (*p* < 0.001) were related to the metastasis of the APN. These factors were included in a binary logistic regression model, and the multivariate analysis revealed that tumor size ≥5 cm (OR = 2.32, 95% CI: 1.30–4.13, *p* = 0.004), mucinous adenocarcinoma or signet ring cell carcinoma (vs. poor differentiated adenocarcinoma, OR = 0.19, 95% CI: 0.08–0.45, *p* < 0.001; vs. moderate to well differentiated adenocarcinoma, OR = 0.22, 95% CI: 0.11–0.42, *p* < 0.001), T4 stage (OR = 1.93, 95% CI: 1.05–3.55, *p* = 0.034), N2 stage (OR = 8.86, 95% CI: 4.45–17.65, *p* < 0.001) and EMVI (OR = 1.88, 95% CI: 1.03–3.42, *p* = 0.040) were independent risk factors for the metastasis of the APN (Table 3). We further conducted a detailed statistical analysis of the number and proportion of ALN-positive cases within each high-risk subgroup, as presented in Appendix D Table A1.

### 3.4. Nomogram Development

**Figure 2:**
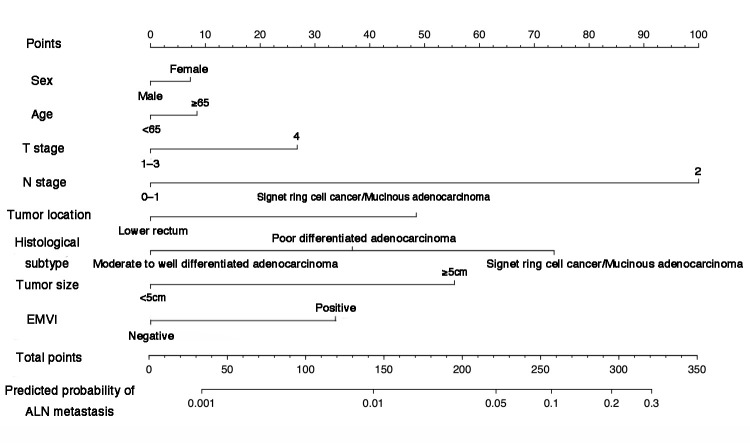
Nomogram for predicting ALN metastasis in rectal and sigmoid colon cancer.

**Figure A2:**
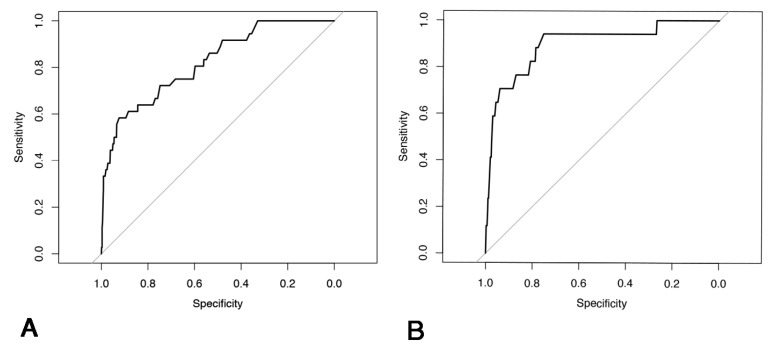
Receiver operating characteristic curve of the nomogram. (**A**) The training set. (**B**) The validation set.

**Figure A3:**
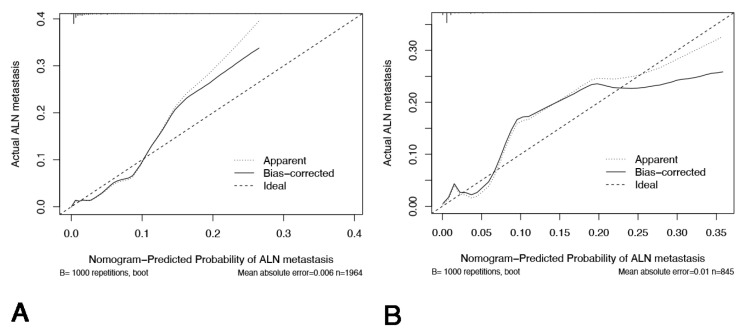
Calibration plot. (**A**) The training set. (**B**) The validation set.

Tumor location, tumor size, degree of differentiation, pathological subtype, T stage, N stage and EMVI were found to be the risk factors for potential ALN metastasis in the univariate logistic regression analysis. These variables were subsequently utilized in the development of a predictive nomogram (Figure 2). The ROC curves are shown in Appendix B Figure A2. The C-index of the nomogram was 0.834 in the training set and 0.890 in the validation set (Appendix E Table A2). Calibration plots are shown in Appendix C Figure A3, and the nomogram was moderately calibrated. The predicted probability of ALN metastasis by the nomogram aligns with the actual occurrence.

The nomogram was constructed based on multivariate logistic regression analysis. Each predictor variable is assigned a point value by drawing a vertical line from the variable to the “Points” axis. The total points correspond to the predicted probability at the bottom of the scale. Variables included in the model were Sex, Age, T stage, N stage, Tumor location, histological subtype and EMVI.

## 4. Discussion

The role of ALN metastasis as a prognostic factor in colorectal cancer remains a topic of debate. Research has yielded different findings on the prognostic significance of ALN metastasis in node-positive rectal and sigmoid colon cancer. Some investigations have identified ALN metastasis as an adverse prognostic indicator [14,15,16,17,18], whereas others have not found it to significantly impact prognosis [5,19]. The 4th edition of the American Joint Conference on Cancer (AJCC) and the TNM Committee of the International Union Against Cancer (UICC) identified apical-node metastasis as N3, indicating its significance [20]. However, subsequent revisions to the TNM classification have removed the N3 category, following evidence indicating a lack of survival disparity between patients classified as having N2 and N3 tumors [21]. As the largest retrospective study to date on ALN metastasis in rectal and sigmoid colon cancer, this finding indicated that the prognosis for colorectal cancer with ALN metastasis was significantly worse compared to patients in TNM stage IIIC and those with N2 stage disease. Additionally, within the population with lymph node metastasis, ALN positivity was an independent predictor of poor prognosis.

Given the close association between ALN metastasis and poor prognosis, formulating an appropriate treatment strategy becomes crucial, with surgical dissection playing a vital role in the overall treatment approach. Nevertheless, there is still considerable controversy regarding the dissection of ALN. Thorough dissection of the ALN at the root of the IMA unequivocally secures an R0 resection and augments the yield of lymph nodes. However, some large cohort studies or meta-analyses have shown that dissection of ALN does not confer additional survival benefits [9,22,23]. This might be attributed to the relatively low occurrence rate of ALN metastasis [24]. Previous research indicates that the prevalence of ALN metastasis varies between 0.3% and 13.9% [11,14,25,26]. In the context of this study, the metastasis rate in the overall cohort was only 1.9%, and even within the TNM stage III population, it was just 4.3%. Therefore, for the vast majority of patients, excessive dissection of ALN appears overly aggressive and not necessary. Not only does it increase the complexity of the surgery, but it also carries a greater risk of damaging the nerve plexus around the IMA root, which can result in disorders of the autonomic nervous system [11,12,27].

If the risk factors for ALN metastasis can be identified through preoperative clinical and pathological data, enabling the prediction of ALN metastasis risk, it would be possible to achieve precise, selective dissection of ALN. Previous studies have indicated that factors such as younger age, advanced T category, advanced N category, higher tumor location, tumor size, pathological subtype, poor differentiation, and positive lymphovascular or perineural invasion might be associated with ALN metastasis [2,11,12,13,14,18,28,29]. However, the relatively limited sample size in these studies, especially those with positive ALN, was inadequate to ensure robust statistical power, thereby yielding inconsistent findings. This limitation results in the current inability to effectively assess the risk of ALN metastasis preoperatively. This study, based on a large cohort, conducted univariate and multivariate analysis on clinical and pathological factors potentially associated with ALN metastasis. It revealed that tumor size ≥5 cm, a T4 stage classification, an N2 stage classification, specific pathological subtypes, such as mucinous adenocarcinoma or signet ring cell carcinoma and EMVI were independent risk factors contributing to the ALN metastasis. Integrating these risk factors with preoperative imaging findings and intraoperative exploration allows for the identification of patients at high risk for ALN metastasis. Selective dissection during surgery, while ensuring the radical resection, can prevent excessive invasion, reduce postoperative complications, and align with the surgical, oncological, and functional principles of modern colorectal cancer surgery.

The metastatic rate of ALNs is relatively low, and most D3 dissections may represent overtreatment. The NCCN guidelines do not clearly define the extent of lymph node dissection for colorectal cancer. It emphasizes en bloc resection of the tumor along with its regional draining lymph nodes and recommends examining at least 12 lymph nodes to ensure accurate N staging. The JSCCR guidelines state that ALN dissection is not routinely indicated for tumors staged cT2 or below, whereas it is routinely recommended for tumors staged cT3/T4. Although the JSCCR provides indications for ALN dissection, formulating a lymph node dissection strategy based solely on clinical T stage is overly simplistic. The findings of this study allow for more nuanced, data-driven surgical decision-making, particularly in cases where the indication for ALN dissection is unclear based solely on conventional guidelines.

Although this study is based on a large multicenter cohort, it still has several limitations. Firstly, as a retrospective study, it suffers from missing some laboratory and radiological data. The predictive model was constructed incorporating only clinical and pathological factors, which impacts the model’s effectiveness. Secondly, although the total number of patients included was large, the incidence of ALN metastasis was relatively low, with only 53 ALN-positive cases (1.9%). This small number may limit the statistical power of subgroup analyses and raises concerns about potential overfitting in the development of the nomogram. This limitation may reduce the robustness of the model and affect its generalizability to broader patient populations. Thirdly, variability in the extent of ALN dissection across different institutions may introduce discrepancies between the gathered data and the actual clinical scenarios. In addition, all enrolled patients did not receive neoadjuvant therapy. As a result, some locally advanced cases, such as those with positive circumferential resection margin (CRM) status, were excluded, leading to the absence of important radiological variables in the predictive model. More importantly, this limits the generalizability of our findings to patients who have received neoadjuvant therapy. Finally, functional outcomes and quality-of-life data, such as bowel, urinary, or sexual function following ALN dissection, were not collected in this study. These outcomes are highly relevant to surgical dissection of ALN but remain unassessed in this study. Given these considerations, prospective studies with larger sample sizes are required to further validate the conclusions of this research.

## 5. Conclusions

The incidence of ALN metastasis in rectal and sigmoid colon cancer is relatively low, yet it significantly worsens prognosis. Independent risk factors for ALN metastasis include tumor size ≥5 cm, classification as T4 stage, classification as N2 stage, histological subtypes of mucinous adenocarcinoma or signet ring cell carcinoma and radiologic evidence of EMVI. Integrating these risk factors with a nomogram predictive model can guide surgeons in performing selective dissection of ALN, achieving radical treatment while avoiding excessive surgical intervention.

## Figures and Tables

**Table 1:** Clinicopathological characteristics.

Variables	N = 2809
Age	61.60 ± 11.19
Gender	
Male	1721 (61.3%)
Female	1088 (38.8%)
Location	
Sigmoid colon	967 (34.4%)
Higher rectum	1198 (42.7%)
Lower rectum	644 (22.9%)
Histological subtype	
Signet ring cell cancer/Mucinous adenocarcinoma	410 (14.6%)
Poorly differentiated	494 (17.6%)
Moderate to well differentiated	1905 (67.8%)
T stage	
1	179 (6.4%)
2	544 (19.4%)
3	1233 (43.9%)
4	853 (30.4%)
N stage	
0	1581 (56.4%)
1	413 (14.7%)
2	815 (29.0%)
TNM stage	
1	554 (19.7%)
2	1027 (36.6%)
3	1228 (43.7%)
Tumor size	
Less than 5 cm	2043 (72.7%)
More than 5 cm	766 (27.2%)
Apical lymph node	
Positive	53 (1.9%)
Negative	2756 (98.1%)
EMVI	
Positive	681 (24.2%)
Negative	2128 (75.8%)
BRAF V600	
Wild-type	2624 (93.4%)
Mutant	185 (6.6%)
Mismatch repair	
Proficient	2577 (91.7%)
Deficient	232 (8.3%)
Adjuvant therapy	
No	859 (30.6%)
Yes	1950 (69.4%)

**Table 2:** Cox regression analysis for risk factors for OS and CSS in the stage III patients.

	OS			CSS		
Variable	HR	95% CI	*p*	HR	95% CI	*p*
Gender						
Male	1.00			1.00		
Female	1.13	0.93 to 1.37	0.228	1.18	0.97 to 1.45	0.100
Age						
<65	1.00			1.00		
≥65	1.06	0.87 to 1.28	0.582	1.04	0.85 to 1.28	0.678
Tumor location						
Rectum	1.00			1.00		
Sigmoid colon	1.23	0.97 to 1.56	0.089	1.27	0.99 to 1.62	0.058
Histological subtype						
Signet ring cell cancer/Mucinous adenocarcinoma	1.00			1.00		
Poor differentiated adenocarcinoma	0.87	0.65 to 1.16	0.336	0.95	0.70 to 1.29	0.752
Moderate to well differentiated adenocarcinoma	0.76	0.58 to 0.99	0.040 *	0.78	0.59 to 1.04	0.092
Tumor size						
<5 cm	1.00			1.00		
≥5 cm	0.93	0.75 to 1.16	0.528	0.98	0.78 to 1.22	0.843
T stage						
T1–3	1.00			1.00		
T4	1.11	0.90 to 1.37	0.342	1.11	0.89 to 1.39	0.343
N stage						
N0–1	1.00			1.00		
N2	1.71	1.37 to 2.14	<0.001 *	1.75	1.38 to 2.22	<0.001 *
Perineural invasion						
Negative	1.00			1.00		
Positive	1.25	1.01 to 1.54	0.041 *	1.26	1.01 to 1.56	0.041 *
Vascular invasion						
Negative	1.00			1.00		
Positive	1.13	0.92 to 1.39	0.247	1.13	0.91 to 1.41	0.258
Apical lymph node						
Negative	1.00			1.00		
Positive	2.02	1.39 to 2.95	<0.001 *	2.06	1.40 to 3.03	<0.001 *
Adjuvant therapy						
No	1.00			1.00		
Yes	0.81	0.43 to 1.52	0.501	1.08	0.51 to 2.30	0.840

* indicates statistical significance.

**Table 3:** Univariate and multivariate analysis for risk factors for ALN metastasis.

					Multivariate Analysis	
Variable	Apical Lymph Node (−) n = 2756	Apical Lymph Node (+) n = 53	Univariate *p*	OR	95% CI	*p*
Age			0.974	—	—	—
<65	1034 (37.5%)	20 (37.7%)				
≥65	1722 (62.5%)	33 (62.3%)				
Gender			0.482	—	—	—
Male	1691 (61.4%)	30 (56.6%)				
Female	1065 (38.6%)	23 (43.4%)				
Tumor location			0.007 *			0.110
Sigmoid/Upper. rectum	2115 (76.7%)	50 (94.3%)		1.00		
Lower rectum	641 (23.3%)	3 (5.7%)		0.42	0.14 to 1.22	
Tumor size (cm)			<0.001 *			0.004 *
<5 cm	2017 (73.2%)	26 (49.1%)		1.00		
≥5 cm	739 (26.8%)	27 (50.9%)		2.32	1.30 to 4.13	
Histological type			<0.001 *			<0.001 *
Signet ring cell cancer/Mucinous adenocarcinoma	386 (14.0%)	24 (45.3%)		1.00		
Poor differentiated adenocarcinoma	485 (17.6%)	9 (17.0%)		0.19	0.08 to 0.44	
Moderate to well differentiated adenocarcinoma	1885 (68.4%)	20 (37.7%)		0.22	0.11 to 0.42	
T stage			<0.001 *			0.034 *
T1–3	1933 (70.1%)	23 (43.4%)		1.00		
T4	823 (29.9%)	30 (56.6%)		1.93	1.05 to 3.55	
N stage			<0.001 *			<0.001 *
N0–1	1982 (71.9%)	12 (22.6%)		1.00		
N2	774 (28.1%)	41 (77.4%)		8.86	4.45 to 17.65	
EMVI			<0.001 *			0.040 *
Negative	2104 (98.9%)	652 (95.7%)		1.00		
Positive	24 (1.1%)	29 (4.3%)		1.88	1.03 to 3.42	
BRAF V600			0.776	—	—	—
Wild-type	2575 (93.4%)	49 (92.5%)				
Mutant	181 (6.6%)	4 (7.5%)				
Mismatch repair			0.443	—	—	—
Proficient	2530 (91.8%)	47 (88.7%)				
Deficient	226 (8.2%)	6 (11.3%)				

* indicates statistical significance.

## Data Availability

The datasets used and/or analyzed during the current study are available from the corresponding authors on reasonable request.

## Abbreviations

ALN	Apical lymph node
EMVI	Extramural venous invasion
CRM	Circumferential resection margin
OR	Odd ratio
CI	Confidence interval
IMA	Inferior mesenteric artery
HL	High ligation
CME	Complete mesocolic excision
TME	Total mesorectal excision
OS	Overall survival
CSS	Cancer-specific survival

## Appendix D

**Table A1:** Proportion of ALN-positive patients across different risk factors.

Risk Factors	T4	N2	Tumor Size > 5 cm	Sigmoid/Upper Rectum	Mucinous Adenocarcinoma/Signet Ring Cell Carcinoma	Vascular or Lymphatic Invasion
Total patients	855	815	766	2164	410	1083
ALN (+) patients	32	41	27	49	24	36
ALN (+) %	3.7%	5.0%	3.5%	2.3%	5.9%	3.3%

## Appendix E

**Table A2:** Relevant parameters of the nomogram.

	C-Index	Sensitivity	Specificity	PPV	NPV
Training set	0.834	0.639	0.93	0.146	0.993
Validation set	0.890	0.882	0.781	0.077	0.997

PPV: Positive predicted value. NPV: Negative predicted value.
